# Case report—malignant transformation in Cronkhite–Canada syndrome polyp

**DOI:** 10.1097/MD.0000000000006051

**Published:** 2017-02-10

**Authors:** Ye Zong, Haiying Zhao, Li Yu, Ming Ji, Yongdong Wu, Shutian Zhang

**Affiliations:** Department of Gastroenterology, Beijing Friendship Hospital, Capital Medical University, Beijing, China.

**Keywords:** case report, Cronkhite–Canada syndrome, endoscopy, malignant transformation, treatment

## Abstract

**Rationale:**

Cronkhite–Canada syndrome (CCS) is a rare disease, the etiology of CCS is currently unknown. Although CCS is widely accepted as a benign disorder, the malignant potential of the polyps in CCS patients is yet controversial.

**Patient concerns:**

A 55-year-old Chinese male was first admitted to Beijing Friendship Hospital with a 3-month history of frequent watery diarrhea (10–15 times/day), loss of taste, and a weight loss of 10 kg in August 2010. The left heel bone fracture in the patient occurred about 2 weeks prior to his diarrhea.

**Diagnoses:**

He was diagnosed as Cronkhite–Canada syndrome.

**Interventions:**

Oral administration of prednisone was initiated at a dosage of 20 mg/day.

**Outcomes:**

After 3 months of treatment, the clinical manifestations disappeared, and colonoscopy showed sparsely distributed small polyps in the colon. Consequently, the dose of prednisone was reduced to 10mg. However, after 4 months, his fingernails were again found atrophic along with mild abdominal discomfort without diarrhea. Colonoscopy revealed a recurrence of the polyps in March 2011. The treatment was repeated with prednisone at a dosage of 20 mg/day resulting in subsided symptoms. In September 2011, he underwent colonoscopy although no significant clinical manifestations were observed. In addition, the polyp in the sigmoid colon was cancerated.

**Lessons:**

The present case indicated that the physical stress was related to CCS and malignant transformation occurred in Cronkhite–Canada syndrome polyp. After the diffused inflammatory polyps have responded to steroid therapy, other existing adenomas require endoscopic treatments, which can decrease the possibility of neoplastic transformation.

## 1 Introduction

The Cronkhite–Canada syndrome (CCS) is a rare disease characterized by the presence of diffused gastrointestinal polyposis, chronic diarrhea, atrophy of the fingernails, cutaneous hyperpigmentation, weight loss, and abdominal pain. However, the etiology of CCS is currently unknown. Although CCS is widely accepted as a benign disorder, the malignant potential of the polyps in CCS patients is yet controversial.^[1]^ Here, we report a case of CCS, which showed that the physical stress was related to CCS and the malignant transformation occurred in the CCS polyp.

## 2 Case report

**Figure 1:**
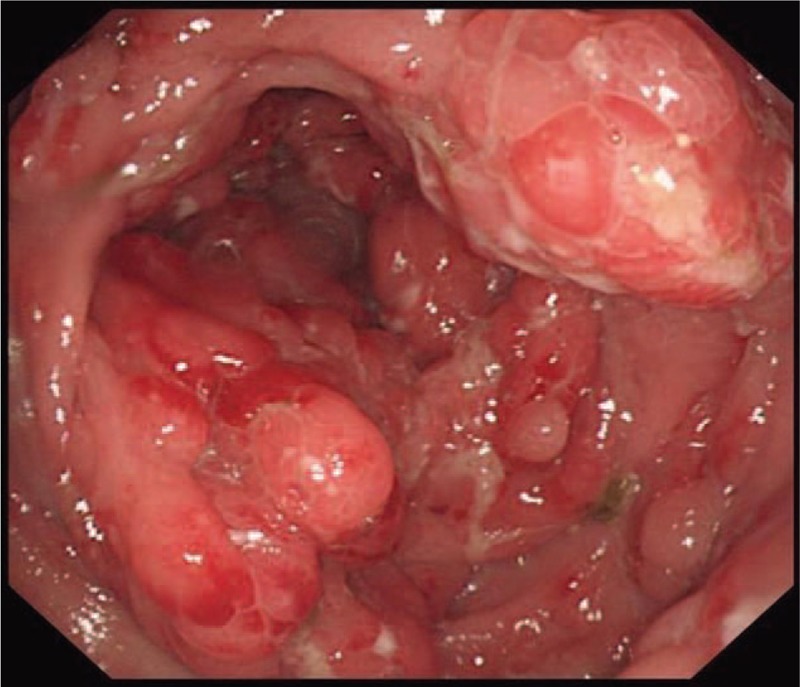
At the first time, the colonoscopy in the patient revealed numerous polyps occupying the colonic and rectal mucosa.

**Figure 2:**
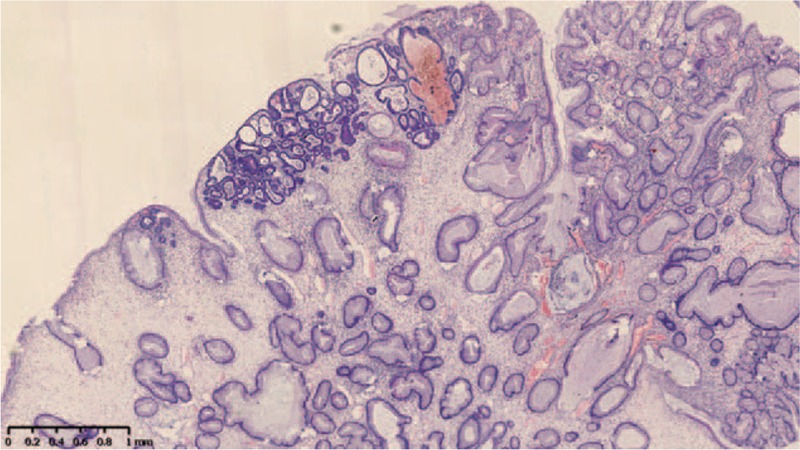
Histological examination of the biopsy specimens obtained from the colon showed adenomas.

**Figure 3:**
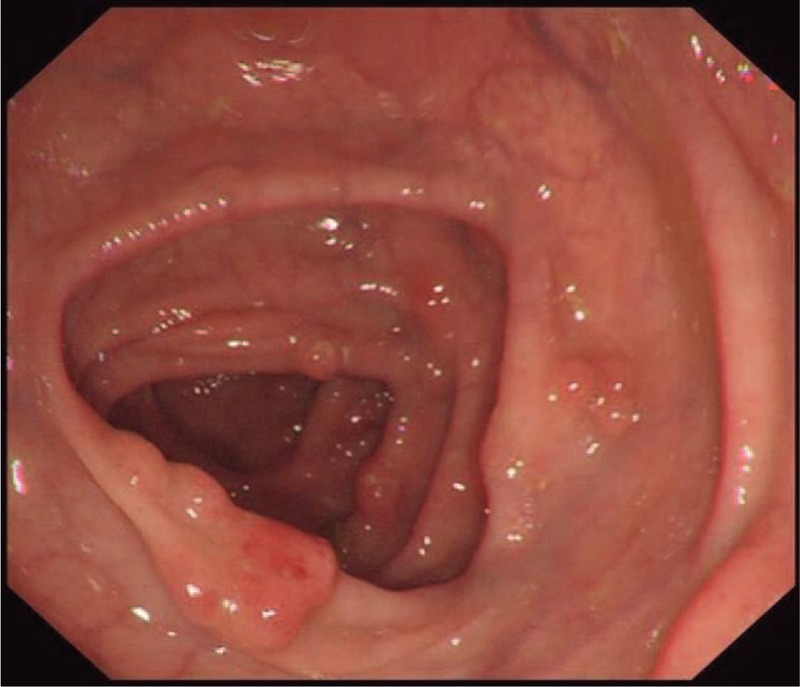
After 3 months of oral treatment with 20 mg prednisone, colonoscopy showed sparsely distributed small polyps in the colon of the patient.

**Figure 4:**
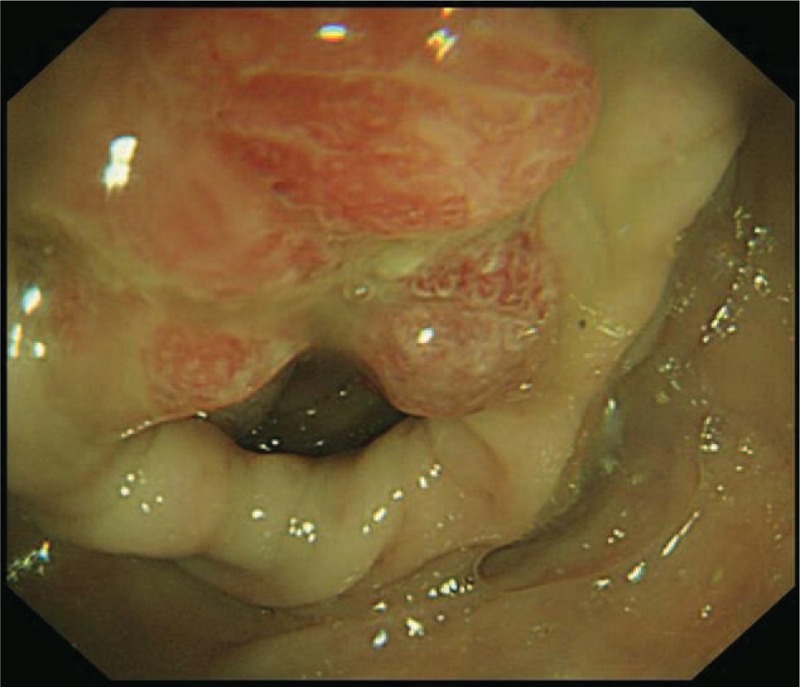
The dose of prednisone was reduced to 10 mg. After 4 months, the colonoscopy revealed the recurrence of the polyps in the patient.

**Figure 5:**
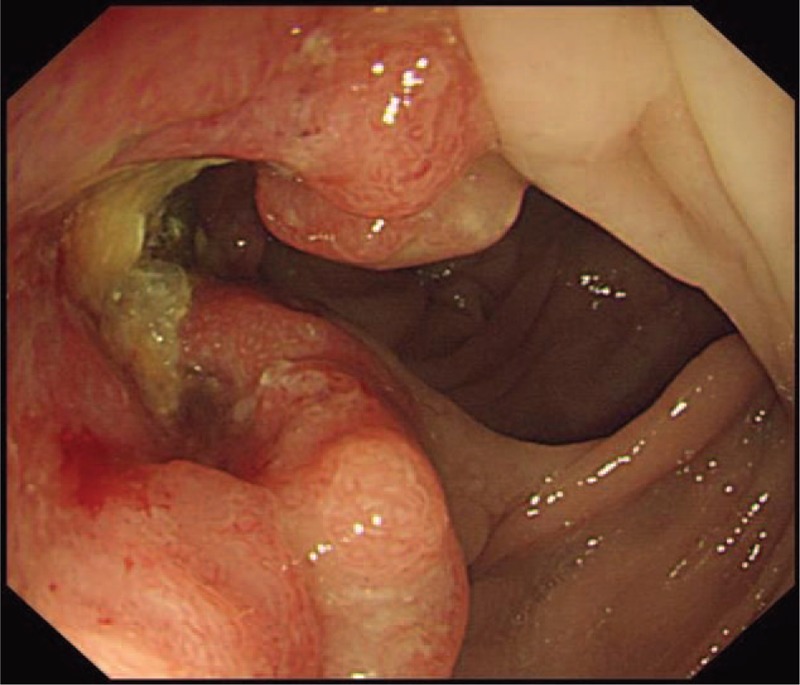
The patient's polyp in the sigmoid colon was cancerated.

**Figure 6:**
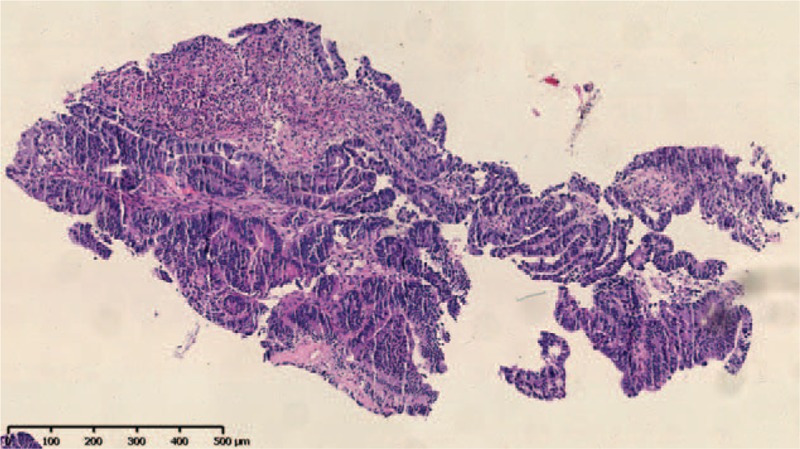
Histological examination of the biopsy specimens obtained from the colon showed adenocarcinoma.

A 55-year-old Chinese male was first admitted to Beijing Friendship Hospital with a 3-month history of frequent watery diarrhea (10–15 times/day), loss of taste, and a weight loss of 10 kg in August 2010. His left heel bone fractured about half a month prior to diarrhea. Neither the patient nor the family reported any history of the gastrointestinal disease. A physical examination revealed a thin man with marked alopecia, brownish macular pigmentation over the palms and soles, and onychodystrophy of the fingernails and toenails. The laboratory findings included serum total protein, 37.1 g/L (normal range 60–80 g/L); albumin, 19.6 g/L (normal range 35–55 g/L); hemoglobin, 10.1 g/dL (normal range 11–16 g/dL); total cholesterol, 2.52 mmol/L, (normal range 3.9–5.2 mmol/L); serum calcium 1.67 mmol/L (normal range 2.0–2.5 mmol/L); and the antinuclear antibody (ANA) was negative. Colonoscopy revealed numerous polyps occupying the colonic and rectal mucosa (Fig. 1). Gastroscopy revealed multiple polyps in the stomach and duodenum similar in appearance to those in the colon and the rectum. A small bowel series showed many filling defects throughout the small bowel, which indicated multiple polyps in the small bowel. Histological examination of the biopsy specimens obtained from the colon and the stomach showed adenomatous (Fig. 2) and inflammatory polyp. Thus, a diagnosis of CCS was made. Oral administration of prednisone was initiated at a dosage of 20 mg/day. Other treatment included oral calcium carbonate 1.5 g and vitamin D3 125U. every day in order to prevent osteoporosis. There were no special requirements on diet. After 3 months of treatment, his clinical manifestation disappeared, and colonoscopy showed sparsely distributed small polyps in the colon (Fig. 3). As a result, the dose of prednisone was reduced to 10 mg/day. After 4 months, his fingernails were again atrophic in addition to mild abdominal discomfort without diarrhea. Colonoscopy demonstrated the recurrence of the polyps (Fig. 4) in March 2011. Subsequently, he was re-administered with prednisone, 20 mg/day. However, the symptoms soon subsided. In September 2011, he underwent colonoscopy although no significant clinical manifestations were recorded. Moreover, the polyp in the sigmoid colon was cancerated (Figs. 5 and 6), following which, he underwent surgery. After surgery, he has been treated with prednisone, 5 mg/day for 5 years. Every 6 months, he underwent colonoscopy, which showed a few polyps which were removed by endoscopic mucosal resection.

## 3 Discussion

The CCS was first described in 1955 by Cronkhite and Canada ^[2]^ as a rare noninherited disorder manifested by diffused gastrointestinal polyposis associated with ectodermal changes, including alopecia, hyperpigmentation, and onychodystrophy. Since then, approximately 400 cases of CCS have been reported worldwide, especially from Japan.^[3]^

However, the etiology of CCS is yet unknown. Here, we reported a case that developed symptoms of CCS after the occurrence of heel bone fracture, which may be one of the causative factors for this symptom. Murata et al reported 1 such case occurring under emotional stress,^[4]^ and Goto^[5]^ indicated that mental and physical stress were confirmed as among the most important risk factors for this syndrome. In the largest single-center series reported on CCS to date, the IgG_4_ infiltration of CCS polyps suggests the possibility of an autoimmune inflammatory process.^[6]^ The positive ANA in some cases and the associated autoimmune diseases such as rheumatoid arthritis and systemic lupus erythematosus in several patients also suggest an autoimmune response, which may be the pathogenic factors for CCS.^[7]^ In the current case, ANA was negative, and the patient did not report any autoimmune diseases. However, his symptoms achieved remission with corticosteroids, which indicated that the autoimmune response was associated with the disease in this patient.

Whether the malignant lesions arise from preexisting polyps or coincidentally is yet controversial. However, in the current case, we found the colon cancer arose from the preexisting polyp. After the first treatment, the polyps of the patient disappeared rapidly but recurred when the dose of prednisone was reduced. The prednisone treatment was considered efficient, and hence, a biopsy sample was not withdrawn ignoring the possibility of neoplastic transformation. Therefore, the conclusions from the current case are: (1) malignant transformation of CCS polyps may occur, the risk of colorectal cancer may warrant aggressive screening in CCS patients, and endoscopic surveillance is vital every 6 months. (2) After the diffused inflammatory polyps have responded to steroid therapy, other existing adenomas require endoscopic treatments, which can decrease the possibility of neoplastic transformation.
